# Editorial: Empowering suicide prevention efforts with generative AI technology

**DOI:** 10.3389/fpsyt.2025.1643893

**Published:** 2025-08-26

**Authors:** Inbar Levkovich, Zohar Elyoseph, Sean Lauderdale, Gunther Meinlschmidt, Bénédicte Nobile, Dorit Hadar Shoval, Yossi Levi-Belz, Shiri Shinan-Altman, J. P. Grodniewicz

**Affiliations:** ^1^ Faculty of Education, Tel Hai College, Upper Galilee, Kiryat Shmona, Israel; ^2^ The Faculty of Education, The University of Haifa, Haifa, Israel; ^3^ Department of Psychology, University of Houston – Clear Lake, Houston, TX, United States; ^4^ Clinical Psychology and Psychotherapy – Methods and Approaches, Department of Psychology, Trier University, Trier, Germany; ^5^ Department of Digital and Blended Psychotherapy and Psychosomatics, Psychosomatic Medicine, University Hospital and University of Basel, Basel, Switzerland; ^6^ Department of Emergency Psychiatry and Acute Care, Lapeyronie Hospital, Montpellier, France; ^7^ Institute of Functional Genomics, Hôpital La Colombière, University Montpellier, Montpellier, France; ^8^ Department of Psychology, Max Stern Yezreel Valley College, Ramat Gan, Israel; ^9^ The Lior Tsfaty Center for Suicide and Mental Pain Studies, Ruppin Academic Center, Emek Hefer, Israel; ^10^ The Louis and Gabi Weisfeld School of Social Work, Bar-Ilan University, Ramat Gan, Israel; ^11^ Copernicus Center for Interdisciplinary Studies, Jagiellonian University, Kraków, Poland

**Keywords:** suicide prevention, generative AI (GenAI), large language models (LLM), risk assessment, mental health, psychosocial autopsy, digital psychiatry

Suicide claims approximately 746,000 lives each year, ranking among the leading causes of premature mortality and psychological distress worldwide (1). Precision risk assessment is especially challenging for high-vulnerability groups, such as military veterans, middle-aged men, and LGBTQ+ individuals. Additional challenges are posed by multiple aspects of stigma (internalized, anticipated, and public), which impedes help-seeking behaviors (2). Stigma of all varieties is driven by the growing influence of media, including user-generated content, which dramatically impacts public perceptions of suicide (3, 4).

In recent years, the rapid advancement of Artificial Intelligence (AI), particularly Generative AI and the large language models (LLMs) that power it, has opened new avenues for suicide risk assessment, prevention, and intervention. Emerging evidence suggests that these technologies can contribute to more personalized and scalable screening tools, enhance the training of mental health professionals, reduce stigma, and support early detection in clinical and digital environments (5, 6). Researchers have also explored how cultural context can influence AI’s sensitivity to suicide risks (7) and how clinical variables such as history of depression, previous suicide attempts, or access to weapons can be integrated into AI models to improve prediction accuracy (5).

The Research Topic brings together multidisciplinary contributions that investigate how Generative AI technologies can be ethically and effectively harnessed to improve suicide prevention. The included articles offer diverse perspectives on technological applications, clinical insights, and ethical considerations with the aim of promoting evidence-based innovation in one of the most urgent areas of mental health.

The articles included in this Research Topic demonstrate the multidisciplinary potential of Generative Artificial Intelligence (GenAI) and large language models (LLMs) to advance suicide prevention. The researchers apply these technologies across diverse contexts, including risk assessment, professional training, public health monitoring, and qualitative analysis, leveraging machine learning to identify previously overlooked risk factors, improve diagnostic accuracy, and support complex clinical decision-making.

The study by Lissak et al. highlights boredom, particularly disengaged boredom, as a significant risk factor for suicide. This conclusion was reached through a hybrid approach that combined large-scale natural language processing with validated psychological measures. Lauderdale et al. examined the ability of three GenAI systems to assess suicide risk among U.S. military veterans. Although the models showed some alignment with clinical judgments regarding chronic risk, they tended to recommend more intensive interventions and displayed greater variability in evaluating acute risk. Zheng et al. developed a predictive model based on comprehensive epidemiological data from the SEER database, identifying heightened risk among older men, residents of rural areas, and patients diagnosed with acute myeloid leukemia. Lastly, Balt et al. evaluated the performance of the Llama 3 model in deductive coding of interviews with individuals bereaved by suicide. While the model demonstrated strong capacity for identifying central themes, the authors also reported concerns related to conceptual inaccuracies and overgeneralizations.

Together, these studies illustrate both the promise as well as the complexity of integrating GenAI into suicide prevention efforts. They emphasize the need for ongoing refinement of AI models, close collaboration with clinical professionals, and the application of ethical frameworks that ensure responsible, context-sensitive, and human-centered implementation.

*Future Directions: Needs, Opportunities, Challenges, and Perspectives*

Generative AI holds transformative potential for suicide prevention, but progress demands a multidisciplinary, ethically grounded approach. As highlighted in several articles in this Research Topic, there is an urgent need to enhance model transparency, interpretability, and contextual sensitivity in both clinical and cultural settings (7, 8). These technologies should be viewed not as replacements for clinical expertise, but as supportive tools that assist in making ethically grounded and context-aware decisions.

There are clear opportunities in developing personalized interventions, emotionally rich training simulations, and novel methods for detecting hidden suicide risk factors. For instance, boredom was identified as a central predictor of suicidality (Lissak et al.), while other studies demonstrated GenAI’s capacity to assess risk in high-vulnerability populations such as veterans (Lauderdale et al.) and leukemia patients (Zheng et al.). In parallel, Generative AI systems may help reduce stigma and encourage help-seeking behavior in marginalized communities (5).

Nevertheless, important challenges remain. These include algorithmic bias, digital inequities, and cultural variability in the expression and recognition of distress (9–11). AI-based tools must be designed with careful attention to gender, cultural diversity, and clinical nuance to ensure fairness and relevance across populations.

Meaningful progress in the field will require collaboration across disciplines, involving clinicians, ethicists, computer scientists, and policymakers, to establish ethical and regulatory frameworks that protect human dignity (12). As illustrated in the work of Balt et al., human feedback and iterative review can strengthen the validity of AI outputs and foster user trust.

As technological capabilities continue to expand, the application of Generative AI in suicide prevention must be guided by principles of responsibility, inclusivity, and a sustained commitment to the wellbeing of vulnerable individuals. Its success will depend on thoughtful implementation, ongoing professional oversight, and continuous ethical engagement.

## Conclusion

The contributions to this Research Topic illustrate the transformative potential of Generative AI in suicide prevention. Across quantitative, qualitative, and theoretical frameworks, the included studies demonstrate how LLMs can support more nuanced risk detection, automate complex coding processes, and uncover novel psychological risks. Simultaneously, the limitations of these technologies, ranging from ethical concerns to contextual misinterpretation, reinforce the need for responsible implementation. Collectively, these studies offer a foundation for AI-assisted suicide-prevention tools – tools that must always complement, not replace, expert human judgement, cultural sensitivity, and empirical rigor.
